# Magnetic Resonance Imaging Findings and Their Association with Electroencephalogram Data in Children with Partial Epilepsy

**DOI:** 10.7759/cureus.7922

**Published:** 2020-05-02

**Authors:** Ngo Minh Xuan, Tran Thi Khanh Tuong, Huynh Quang Huy, Nguyen Huu Son

**Affiliations:** 1 Pediatrics, Pham Ngoc Thach University of Medicine, Ho Chi Minh, VNM; 2 Internal Medicine, Pham Ngoc Thach University of Medicine, Ho Chi Minh, VNM; 3 Radiology, Pham Ngoc Thach University of Medicine, Ho Chi Minh, VNM; 4 Pediatrics, Hue Central Hospital, Hue, VNM

**Keywords:** partial epilepsy, magnetic resonance imaging (mri), electroencephalography (eeg)

## Abstract

Background

It is important to identify the neuroimaging features that are associated with partial epilepsy in children. Advances in technology have recently been made to localize focal epileptogenic lesions, especially high-resolution structural imaging with magnetic resonance imaging (MRI). The recommendation that electroencephalography (EEG) should be the gold standard and that MRI should be optional has been questioned. The present study aims to evaluate the efficacy of MRI in children with partial epilepsy and to compare the diagnostic yields of MRI and EEG data.

Methods

The present study was conducted among one hundred twelve 1- to 6-year-old children with partial epilepsy. All patients underwent EEG and brain MRI. The epileptogenic lesions were identified on the basis of the signal intensities and morphological abnormalities seen on MRI. The correlation between MRI and EEG abnormal findings was analyzed using a chi-square test.

Results

Abnormal MRI findings were present in 34.8% (n = 39) of the sample. The EEG and MRI data agreed with respect to classifications into abnormal or normal in 48.2% of the sample (n = 54). Of the 27 patients with normal EEG findings, six (22.2%) had abnormal MRI findings. Inter-rater agreement showed the compatibility between EEG and MRI not significant (weighted Kappa = 0.105).

Conclusion

A number of MRI abnormalities were found in our study of otherwise normal children, although the correlation between these results was not clear. The follow-up of these children will help us identify the important abnormalities. Despite the small sample size, our results showed that normal EEG findings do not predict normal brain MRI data in children with partial epilepsy.

## Introduction

Epilepsy is a disorder of the cerebral cortex in which symptoms occur due to an excessive, abnormal, sudden, synchronous discharge of neurons [1]. This abnormal, sudden brain stimulation is intermittent, usually short-term and self-limiting, lasting from a few seconds to a few minutes. Epilepsy should include the occurrence of at least two unprovoked seizures [2]. Approximately 10.5 million children under age 15 have epilepsy worldwide, accounting for a quarter of all patients with epilepsy. Of these, about 1.12 million children live in developing countries [3]. The prevalence of pediatric patients with epilepsy has been reported to be up to 10% in the literature. The incidence of the disease is 3.6% during the lifetime, and more than half of those starts in childhood [4-6]. Children are considered to have epilepsy when seizures occur repeatedly for a period of time without any apparent reasons. Epilepsy is not a specific disease, but to diagnose epilepsy, other causes of seizures must be eliminated, such as tumors, sclerosis, acute brain injury, hemorrhage, infection, and genetic disease [7].

Partial epilepsy is considered to include epileptogenic seizures and to be the type of epilepsy most resistant to antiepileptic drugs. The leading causes of this form of epilepsy in early childhood are defects acquired during the course of development, hypoxic-ischemic encephalopathy insult, perinatal infections, tuberous sclerosis, and metabolic diseases [8].

Electroencephalography (EEG) and neuroimaging are two common methods used to distinguish, confirm, or reject an epilepsy diagnosis. Researchers and clinicians have also sought to identify the cause of epilepsy to classify epilepsy syndrome. Magnetic resonance imaging (MRI) is more sensitive than computed tomography scans for most brain epileptic lesions [9-10]. EEG is an important and routine way to identify seizures in children. Essentially, each child with repeated seizures should undergo an EEG while awake and asleep.

The purpose of this article is to explore brain lesions via MRI and their relation to electroencephalograms in children with partial epilepsy.

## Materials and methods

Patients

In this prospective study, we consecutively investigated all 1- to 6-year-old children with partial epilepsy who were referred to the Pediatric Center of Hue Central Hospital from January 2018 to December 2019. Patients who have received antiepileptic drugs were excluded. The study was conducted after receiving approval from the Ethical Committee of the hospital under the reference number of 01012018/HCH. Written informed consent was obtained from all the children’s parents or guardians.

We defined partial epilepsy as seizures that affect only one part of the brain clearly proven by clinics and/or EEG data, occurring more than once among children without fever [11], and without any laboratory abnormalities or evidence of acute nervous system disorder. Children with acute cerebral insults were excluded from the study. Demographic information and clinical data included the patients’ age and sex.

EEG data

**Figure 1:**
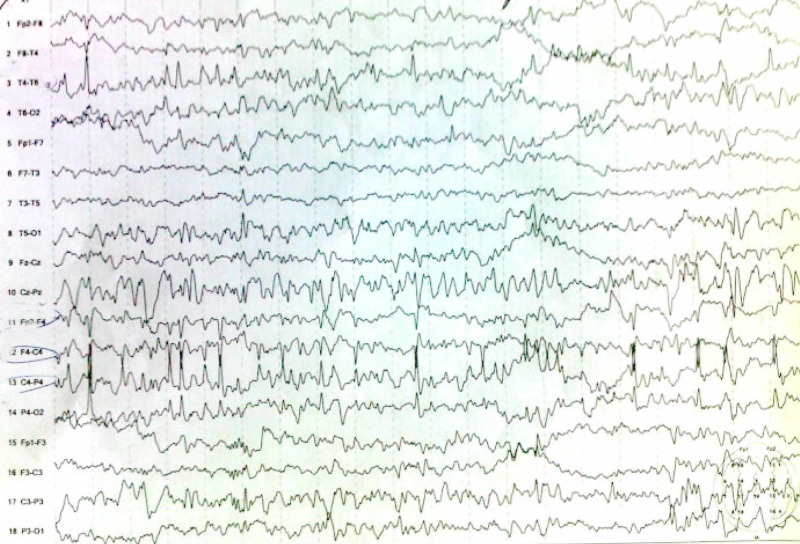
EEG findings of a 6-year-old male patient Interictal EEG showed spikes on the right hemisphere. EEG, electroencephalography

A surface EEG was recorded for all patients. EEG findings were classified as “yes” or “no” for three different criteria: abnormal, epileptiform discharges (Figure 1), and focal slowing. Benign focal EEG variants were not regarded as abnormal [12]. The EEG data were only interpreted by one pediatric neurologist after performing an MRI scan, and the neurologist was blinded regarding the findings.

MRI protocol

The MRI study was performed within the first months of the second seizure whenever possible, and images were acquired on 1.5 Tesla (Siemens, Germany) using a head coil and a gradient strength of 45 mT. The protocol consists of an axial T2 weighted sequence (T2W), a sagittal T1 weighted sequence (T1W), an axial fluid attenuation inversion recovery sequence (FLAIR), diffusion-weighted imaging (DWI) and an axial gradient echo (GRE). A single radiologist who was blind to the clinical features, EEG findings, or any information about the patients interpreted the MRI scans. MRI findings were classified into six categories of lesions: cortical abnormalities, white matter abnormalities, enlarged lateral ventricles, volume loss, encephalomalacia, and a miscellaneous group.

Statistical analysis

SPSS 16 version software (SPSS Inc., USA) was used to analyze the data. Quantitative variables are displayed as the mean (standard deviation). The qualitative variables were expressed in terms of proportion. The chi-square test was used to test for the difference in proportion. The correlation between MRI and EEG findings was explored using the chi-square test and weighted Kappa. A P-value <0.05 was considered statistically meaningful.

## Results

In total, 112 pediatric patients met the inclusion criteria for this ongoing study. There were 58 (51.8%) boys and 54 (48.2%) girls, aged 12 to 72 months old (mean ± SD: 28.0 ± 22.9 months). Eighty-five (75.9%) of the patients had focal epileptiform discharges. Of those, 43 had bilateral independent EEG findings, and 42 had unilateral findings (26 on the left and 16 on the right). MRI abnormalities were present in 39 (34.8%) patients (Table 1).

**Table 1:** Patient’s characteristics, EEG and MRI findings EEG, electroencephalography; MRI, magnetic resonance imaging

Variables	All patients (n = 112)
Age (month)	28.0 ± 22.9
Sex	
Male	58 (51.8%)
Female	54 (48.2%)
Seizure Type	
Simple	13 (11.6%)
Complex	61 (54.5%)
Secondary generalization	38 (33.9%)
EEG epileptiform discharges	
Bilateral independent	43 (38.4%)
Unilateral	42 (37.5%)
MRI findings	
Cortical lesion	5 (4.5%)
White matter lesions	10 (8.9%)
Encephalomalacia	1 (0.9%)
Volume loss	2 (1.8%)
Enlarged ventricles	9 (8.0%)
Miscellaneous abnormalities	12 (10.7%)

**Figure 2:**
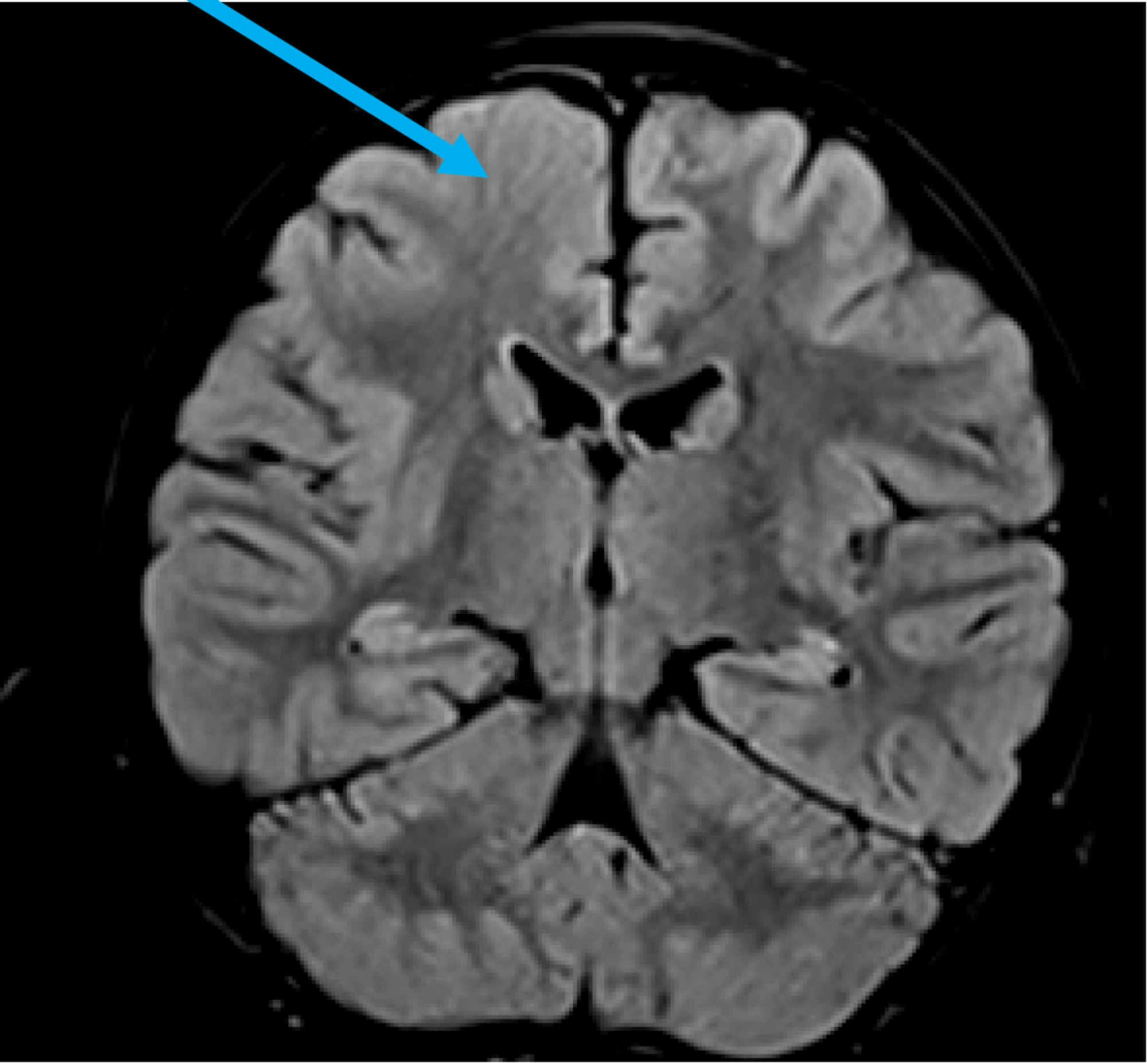
MRI findings of a six-year-old patient showed a focal cortical dysplasia on the right frontal pole (axial plane) MRI, magnetic resonance imaging

**Figure 3:**
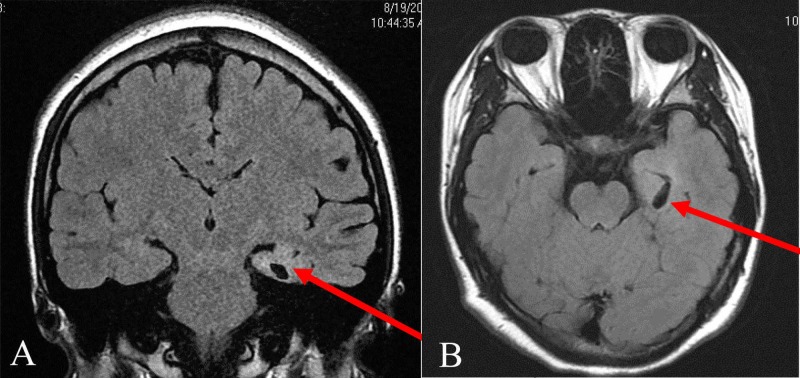
A patient with hippocampal sclerosis on the left temporal lobe. A: coronal plane; B: axial plane

Cortical abnormalities were observed in 5 patients (accounting for 4.5% of the total group), including two cortical dysplasia/heterotopia (Figure 2) and three hippocampal abnormalities (Figure 3).

**Figure 4:**
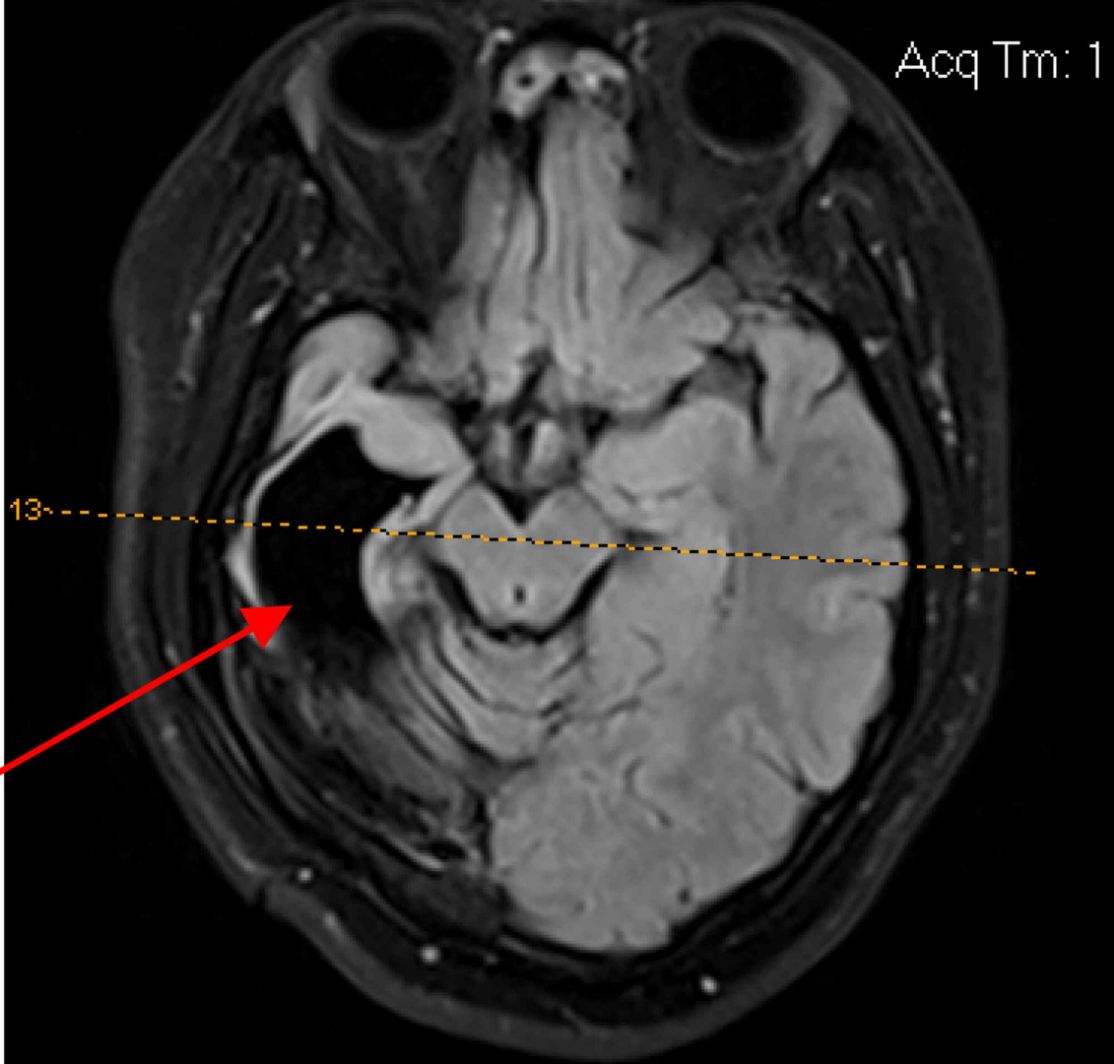
Volume loss in the right hemisphere after intracerebral hemorrhage due to a vitamin K deficiency

**Figure 5:**
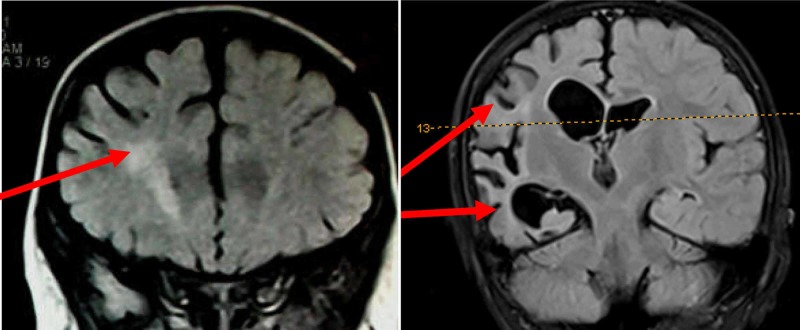
A 4-year-old patient with volume loss and enlarged ventricles after Rasmussen’s encephalitis

Encephalomalacia was found in one child, volume loss was detected in two patients (Figure 4), white matter abnormalities were found in 10 patients, and enlarged ventricles were noted in nine patients. Miscellaneous abnormalities were described in 12 participants (Figure 5).

Both the EEG and MRI findings were abnormal in 33 (29.5%) patients (Table 2).

**Table 2:** Comparison of EEG and MRI findings EEG, electroencephalography; MRI, magnetic resonance imaging

EEG	MRI		
	Normal	Abnormal	Total
Normal	21 (18.7%)	6 (5.4%)	27 (24.1%)
Abnormal	52 (46.4%)	33 (29.5%)	85 (75.9%)
Total	73 (65.2%)	39 (34.8%)	112 (100%)

In addition, 21 patients had normal MRI and normal EEG findings. Among the other cases, MRI findings were classified as normal and EEG findings were classified as abnormal in 52 cases, and MRI findings were classified as abnormal and EEG findings were classified as normal in 6 cases. Of the 27 patients with normal EEG findings, six (22.2%) were found to have an abnormal MRI results. Using a chi-square test, the association between MRI and EEG findings was not significant (*P* = 0.1784). Also, there was not compatible between MRI and EEG findings (weighted Kappa = 0.105).

## Discussion

MRI has been considered to be effective in evaluating the brain structure, as well as epileptogenic lesions and other abnormalities in partial epilepsy in children. MRI is widely accepted as a highly sensitive and noninvasive neuroimaging method. The International League Against Epilepsy recommended performing diagnostic imaging for children when localization-related new-onset epilepsy is not known or in question, when epileptic syndrome with unlikely symptomatic etiology is present or when an epilepsy classification is suspected. Magnetic resonance is preferred to computed tomography because of its versatility, superior resolution and lack of radiation exposure to patients [13].

Approximately 15% to 30% of patients with partial epilepsy do not respond to antiepileptic drugs; this matter needs to be evaluated with neuroimaging studies to identify possible structural abnormalities that might be responsible for seizures [14-15]. Detection of a structural substrate via MRI should be considered for further management as a patient’s opportunity to be considered for surgical treatment is greatly enhanced when a structural abnormality is found by MRI [16]. Approximately 60% of patients become seizure-free after an operation, and the seizure-free post-surgical outcome is 75% for neoplasms, 67% for hippocampal sclerosis, and 58% for cortical dysplasia [17].

Approximately half of the sporadic imaging studies in pediatric patients with new-onset epilepsy associated with localized epilepsy have been reported to be abnormal; 15% to 20% of imaging studies obtained useful information on the causes and/or seizure focus, and 2% to 4% obtained information that could modify urgent medical management [13]. On the other hand, more than 5% of the scans performed on healthy adults showed occasional findings. Several studies have shown the prevalence of abnormal neuroimaging results in pediatric patients with acute seizures. Abnormal neuroimaging detection rates in these studies ranged from 0 to 38.6% [10,18-23].

Our study analyzed 112 children 1 to 6 years of age with partial epilepsy, and abnormal magnetic resonance results were observed in 39 (34.8%) patients, which is more than that observed in other studies. This difference may be due to abnormal findings detected by magnetic resonance that have a significant association with partial epilepsy [24-25]. According to Kast, the MRI findings of patients presenting with partial epilepsy who underwent focal seizure protocol imaging showed that the detection rate of abnormalities was high with partial epilepsy [26]. Previous studies of both partial and generalized types of seizures have found a lower incidence of neuroimaging abnormalities [23,27]. Other pediatric investigations have recommended that partial epilepsy is more often correlated with abnormal neuroimaging, which is concordant with our study [28-29].

Kalnin et al. reported the frequency of MRI abnormalities in children with a first recognized seizure of approximately 31% [30]. Of these, leukomalacia and ventricular enlargement were the most common abnormalities observed in this study, which was similar to our results. The epileptogenic lesions, which include hippocampal sclerosis, infections, gliosis, tumors, vascular malformations, and developmental anomalies, were identified on the basis of the MRI features typical for each of these abnormalities. A diagnosis of hippocampal sclerosis was based on the principal findings of volume loss and an abnormal signal in the hippocampus with the loss of hippocampal architecture on inversion recovery sequence images. In our study, hippocampal sclerosis was observed in 3 (2.7%) patients, which was lower than that observed in other reports.

We investigated EEG abnormalities and epileptogenic lesions via MRI. Although the sample size in our study was small, we found that there was no correlation between MRI and EEG findings. This means that normal EEG findings could not predict a normal MRI scan. These results suggest that normal findings on EEG should not be used to put an epileptic patient at low risk and these patients should undergo an MRI for further evaluation. Follow-up is necessary to detect the significant abnormalities found on neuroimaging.

Our study recommends a need to keep exploring neuroimaging techniques in pediatric patients with partial epilepsy, which is consistent with the suggestions of the clinical approach for assessing the first nonfebrile seizure in pediatric patients. It also provides valuable evidence for cohorts with a large number of pediatric patients who can be followed through their developmental process and seizure recurrence. Additional assessments through EEG and neuroimaging may show progressive changes and allow retrospective risk analysis.

## Conclusions

We found a number of neuroimaging abnormalities in pediatric patients with partial epilepsy. Information obtained might help guide surgical or medical management. Although EEG and MRI are the best diagnostic tools for an accurate diagnosis in a pediatric patient presenting with the first episode of seizure, our findings did not show the correlation between EEG and neuroimaging findings and demonstrated that EEG data should not be used as the only criterion for a subsequent MRI.
